# Validation and adaptation of the diffusion of intimate images among adolescents (EDIMA) scale in adolescents from Peru

**DOI:** 10.3389/fpsyg.2024.1399423

**Published:** 2024-12-16

**Authors:** Joel Figueroa-Quiñones, Miguel Ipanaqué-Zapata, Oriana Rivera-Lozada, Cecilia Gabriela Villalobos-Quiroz, Axel Glynda Vilcabana-Burgos, Ibeth Solange Valle-Chafloque

**Affiliations:** ^1^Universidad Autonoma de Ica, Ica, Peru; ^2^Universidad Señor de Sipán, Chiclayo, Perú; ^3^Asociación de estudiantes de medicina de la Universidad Señor de Sipán (ASOCIEM USS), Chiclayo, Peru

**Keywords:** adolescents, reliability, psychometrics, intimate images, EDIMA

## Abstract

**Background:**

The use of technologies through social networks is a common practice among adolescents who use it to communicate. However, the lack of control and supervision of these media means that they disseminate any type of information, including sexual content.

**Objective:**

To evaluate the psychometric properties of the Intimate Images Diffusion Scale (EDIMA) in Peruvian adolescents.

**Method:**

A psychometric study was conducted with a population of 900 adolescents from the coast, highlands and jungle of Peru. A confirmatory factor analysis and measurement invariance analysis was performed by age and sex groups. In addition, internal consistency was evaluated through the omega index to estimate the reliability of the scale.

**Results:**

Our study identified that the two-factor model obtained adequate fit indices with values >0.994 for the CFI and TLI, while the SRMR had a value of 0.074 and the RMSEA of 0.058. Measurement invariance by age group reported a ΔCFI and ΔRMSEA ≤0.01. Reliability reported a McDonald’s *ω* greater than 0.8.

**Conclusion:**

The EDIMA is a valid and reliable instrument to evaluate the dissemination of intimate images among adolescents from various regions of Peru.

## 1 Introduction

Adolescence is a stage of physical, social and psychological changes that lead human beings to adulthood (Gaete, 2015). During this stage, social relationships and interaction with peers are priorities for adolescents, which is why electronic devices such as cell phones, the Internet and social networks have become the most popular media among this population, as they offer ease of socialization activities (Ehrenreich et al., 2021; Subrahmanyam and Greenfield, 2008). However, several studies warn of the dangers to which adolescents are exposed when using mobile devices and surfing the Internet without any control or supervision (Squicciarini et al., 2010; Agarwal and Kar, 2015; De Oliveira et al., 2017).

One of the emerging phenomena in this context is “sexting,” which is defined as sending, receiving and forwarding erotic or sexual content to other people through electronic devices (Cooper et al., 2016). For example, this behavior consists of sending intimate images (messages, photos or videos) to another person with whom the sender does not necessarily maintain an affective bond (Gámez-Guadix et al., 2015; Hasinoff, 2013). On the other hand, it includes the reception of this type of content and the forwarding of intimate images, which often involves dissemination without the consent of the original sender, generating risks of digital aggression and serious privacy violations (Moritz and Christensen, 2020). Each of these dimensions such as sending, receiving and forwarding represent distinct but interconnected roles within the phenomenon of intimate image dissemination, and differentiating them allows capturing the frequency of this behavior and the specific risks that each dimension entails (Penado et al., 2019).

The practice of disseminating intimate images in different countries reveals the frequency in adolescents of different demographic and cultural aspects (Doyle et al., 2021). For example, one study found that the most frequent practice of this behavior was in Colombian adolescents (48.9%) compared to the Spanish population (28.2%) (Gil-Llario et al., 2021). Another study indicated that Spanish adolescents practice sexting more frequently (57.6%) than Americans (40.5%), however, the latter are more likely to receive messages with sexual content (15.0%) (Gassó et al., 2021). And in Brazil a study reported that 20.8% of students practiced sexting and admitted to having sent intimate images of their partner (De Silva et al., 2020).

Globally, there are several instruments used to measure the dissemination of intimate images through social networks. For example, the sexting practice instrument, developed and validated in Mexican youth (Andoney and Montijo, 2021), offers the assessment of feelings and motivations for sexting practices, however, no other analyses have been conducted to confirm the robustness of the proposed structure. Another study, developed a scale to measure sexting behavior among Spanish adolescents (Rodríguez-Castro et al., 2021), although this instrument is brief with 9 items, it does not provide information on the functioning of the items in the groups analyzed, and in Spain, researchers developed the 65-item Adolescent Sexting Scale (A-SextS) to assess this problem (Molla-Esparza et al., 2023), however, it turns out to be too extensive an instrument and could fatigue adolescents. Likewise, one of the most reliable instruments to assess this construct is the Intimate Image Dissemination Scale (EDIMA), proposed by Penado et al. (2019). This study developed the scale with the aim of measuring the behaviors of receiving, sending and forwarding intimate images among adolescents. To evaluate the tool, the authors conducted a confirmatory factor analysis, demonstrating the construct validity of the 20-item scale with adequate fit indices and its internal consistency across Spaniards aged 12 to 19 years. Thus, the EDIMA is established as a solid instrument to investigate the phenomenon of sexting.

Assessment measures need to show that the relationships between items and their factors are equivalent and that characteristics and differences between ages, genders, cultures, etc. of a group do not affect the performance of the instrument. In fact, some studies have shown that the practice of sexting is related to age (Madigan et al., 2018). For example, in the Netherlands a study reports that adolescents aged 15 to 17 years sent more sexual messages compared to those aged 12 to 14 years (Boer et al., 2021). Likewise, in Spain another study revealed that the prevalence of sexting increased 10 times more among older adolescents (Gámez-Guadix et al., 2015). However, all these studies assume the objectivity of their assessments without being certain of their equivalent performance despite age differences (Byrne, 2010). In that sense, the assessment of sexting through factorial invariance allows the conclusions obtained from the instrument to be applicable to different age groups. If factorial invariance is established, it allows researchers to make valid comparisons and obtain reliable conclusions about the factors being measured (Meredith, 1993).

In Peru, a study reported that 20% of adolescents engaged in sexting, of which males doubled the frequency (35%) of this type of practice (West et al., 2014). However, this study used a self-report questionnaire and without psychometric evidence of its validity and reliability, so the results of that study could be biased (West et al., 2014; Luján Tangarife and Cardona Arias, 2015). Likewise, another study with Peruvian adolescents and adults revealed sexting prevalences of 14 to 25% in males and 6 to 13% in females, however, they used a Spanish instrument adapted only through expert judgment and reliability estimated by a pilot test (Vega-Gonzales, 2020). Therefore, it is crucial to have a valid and reliable instrument to measure the construct of dissemination of intimate images among adolescents, since in this way it will be possible to understand the magnitude and consequences of this phenomenon in the Peruvian adolescent population.

The present study in Peruvian adolescents had the following objectives: (a) To confirm the structure of the scale, (b) To evaluate its measurement invariance among subgroups according to age and sex groups, (c) To determine the reliability of the scale.

## 2 Methods

### 2.1 Design

The quantitative study was of an instrumental nature, given that an analysis and adaptation of the EDIMA was carried out.

### 2.2 Population and sample

The population consisted of 900 adolescent students residing in the coast (Chiclayo), highlands (Cajamarca) and jungle (Tarapoto) of Peru, with 300 participants in each region. These regions were selected to ensure representation of the geographic and cultural diversity of the country. Peru is characterized by a heterogeneous geography with differences between the coastal, Andean and Amazonian regions, which influences the experiences and behaviors of its inhabitants. The mean age was 14.3 years with a minimum of 12 and a maximum of 17 years and a SD of 1.35. The proportion of females (*n* = 455) was equivalent to that of males (see Table 1). Prior informed consent was collected from their parents. Finally, Kline (2016) and Schreiber et al. (2006) propose that a sample of at least 200 to 500 participants is adequate for CFA estimates. We applied non-probability convenience sampling by selecting educational institutions that agreed to participate voluntarily to obtain a sample of 900 participants. Therefore, the sample size used in our study exceeds these recommended thresholds.

**Table 1:** Characteristics of the participants and differences in the EDIMA scores.

Characteristics	*F* (%)	*M* (SD)	*p*
Age			
12	90 (10.0)	21.3 (5.1)	0.31
13–14	391 (43.5)	22.6 (7.5)	
15	217 (24.1)	23.0 (8.1)	
16–17	202 (22.4)	22.5 (6.6)	
Sex			
Men	445 (49.4)	22.7 (7.5)	0.72
Women	455 (50.6)	22.5 (7.0)	
Region			
Coast	300 (33.3)	22.8 (8.7)	0.31
Jungle	300 (33.3)	21.5 (5.0)	
Mountains	300 (33.3)	23.4 (7.4)	

F, frequency; %, percentage; M, media; SD, desviación estándar; *p*, *p* value.

### 2.3 Procedures

First, authorization was obtained from the principal investigator of the study, Dr. María Luisa Rodicio García, for the validation of her instrument. Second, the EDIMA was used and 1 focus group was conducted virtually with adolescents (*n* = 6, 3 males and 3 females) for the purpose of examining the comprehension and language used in the questions. A researcher of this study with training in conducting focus groups led the sessions. The session was recorded for analysis. Based on the findings, the items were modified to adapt the language of the instrument to the local context population.

Authorization was requested from each director of the educational institutions in the 3 Peruvian regions to access their facilities. Subsequently, for data collection, 6 psychology students (2 in each city) were trained virtually by the principal investigators of the study, prior to the application of the instrument, on the objectives of the study, the use of the instrument and ethics in research with human beings. The collectors provided informed consent to the parents of the adolescents, detailing the purpose of the evaluations of their children and informing them of the anonymity and confidentiality of the data obtained from the minors. The participants were evaluated in the classrooms of the institutions during regular school hours during the school period in the months of November 2023. Those who met the criteria and gave assent and informed consent were surveyed. The responses obtained were digitized in excel files and were protected with passwords with access only by the principal investigators.

### 2.4 Instruments

The Intimate Image Dissemination Scale among Adolescents (EDIMA) was originally developed by María Penado and her collaborators in Spain during 2019. This instrument measures the frequency of sharing and dissemination of images or texts with sexual content. The scale has 20 items, and distributed in two factors (through mobile chats and through social networks). The scale is a five-point Likert-type scale (1 = never and 5 = always), with a maximum of 100. Regarding its properties, the instrument shows an overall reliability of *α* = 0.976 and *Ω* = 0.981, and a good fit of the model with SRMR ≤0.06 y GFI, AGFI, NFI y RFI > 0.90 (Penado et al., 2019).

### 2.5 Analysis plan

Descriptive statistics were applied to evaluate the items, which were analyzed by means of mean; standard deviation, frequencies and percentages (Byrne, 2010). Likewise, for the difference in means between EDIMA scores and groups, the scores were analyzed in Student’s t-test and Anova for independent samples. Values of *p* < 0.05 were considered statistically significant.

A confirmatory factor analysis (CFA) was used to confirm the structure of the EDIMA. For this purpose, a polychoric matrix and the variance-adjusted weighted least square mean and variance-adjusted least square mean estimator (WLSMV) were applied. Also, a Comparative Fit Index (CFI; >0.95), Comparative Fit Index (TLI; >0.95), root mean squared error of approximation (RMSEA; < 0.06) and standardized root mean squared error (SRMR; < 0.08) were considered as adequate (Byrne, 2010).

The factorial invariance of the EDIMA, as a function of age and sex groups was assessed using MIMIC (Multiple Indicator, Multiple Cause) models and the possibility of invariance was established when ΔCFI ≤0.01 and ΔRMSEA ≤0.015 (Nunnally and Bernstein, 1994).

Reliability was assessed by the omega coefficient (*ω*). When ω was greater than 0.7, it was considered acceptable (Nunnally and Bernstein, 1994). Data were analyzed through the R studio statistical software platform with the packages “lavaan,” “semTools,” and “semPlot.”

### 2.6 Ethics statement

The present study was approved by the Institutional Committee of Ethics in Research of the Catholic University of Los Angeles of Chimbote with resolution n° 0115–2023. In addition, the ethical principles of justice, respect and beneficence in research with human beings were applied (Manzini, 2000). For example, through informed consent applied to parents so that they provide authorization for the participation of their minor children and informed assent. In addition, participants’ digitalized questionnaire responses were protected with unique access passwords for the principal investigators to prevent the identity of the participant and protect their data.

## 3 Results

### 3.1 Descriptive analysis of participants and EDIMA

In the age group, adolescents aged 15 years present a slightly higher mean (*m* = 23.0) compared to the other groups. Likewise, in both sexes, similar means were obtained (22.7 for males and 22.5 for females). In terms of region, adolescents from the mountains showed a higher mean (*m* = 23.4) compared to those from the jungle and coast. However, none of these scores were statistically significant.

### 3.2 Descriptive analysis of the items

The descriptive analysis of the 20 EDIMA items shows that item 1 has the mean score (*M* = 1.416) and the highest variability (SD = 0.795). The item-rest correlation reveals correlations above 0.503 for all items. Likewise, the consistency of item 1 (*ω* = 0.939) was the highest and the lowest was item 18 (*ω* = 0.818) (see Table 2).

**Table 2:** Items analysis for the EDIMA scale.

Items	*M*	SD	Item-rest correlation	*ω*
Item 1	1.416	0.795	0.503	0.939
Item 2	1.133	0.49	0.666	0.932
Item 3	1.154	0.541	0.725	0.930
Item 4	1.118	0.51	0.617	0.933
Item 5	1.196	0.638	0.756	0.928
Item 6	1.119	0.445	0.794	0.929
Item 7	1.109	0.531	0.745	0.931
Item 8	1.143	0.565	0.706	0.930
Item 9	1.206	0.61	0.577	0.934
Item 10	1.103	0.459	0.649	0.934
Item 11	1.124	0.58	0.697	0.933
Item 12	1.077	0.377	0.657	0.933
Item 13	1.134	0.559	0.732	0.930
Item 14	1.099	0.443	0.723	0.932
Item 15	1.089	0.473	0.660	0.934
Item 16	1.087	0.431	0.677	0.933
Item 17	1.111	0.509	0.736	0.841
Item 18	1.069	0.429	0.688	0.818
Item 19	1.070	0.430	0.631	0.832
Item 20	1.074	0.406	0.695	0.872

M, mean; SD, standard deviation; ω, coefficient of omega.

### 3.3 Confirmatory factor analysis

**Figure 1:**
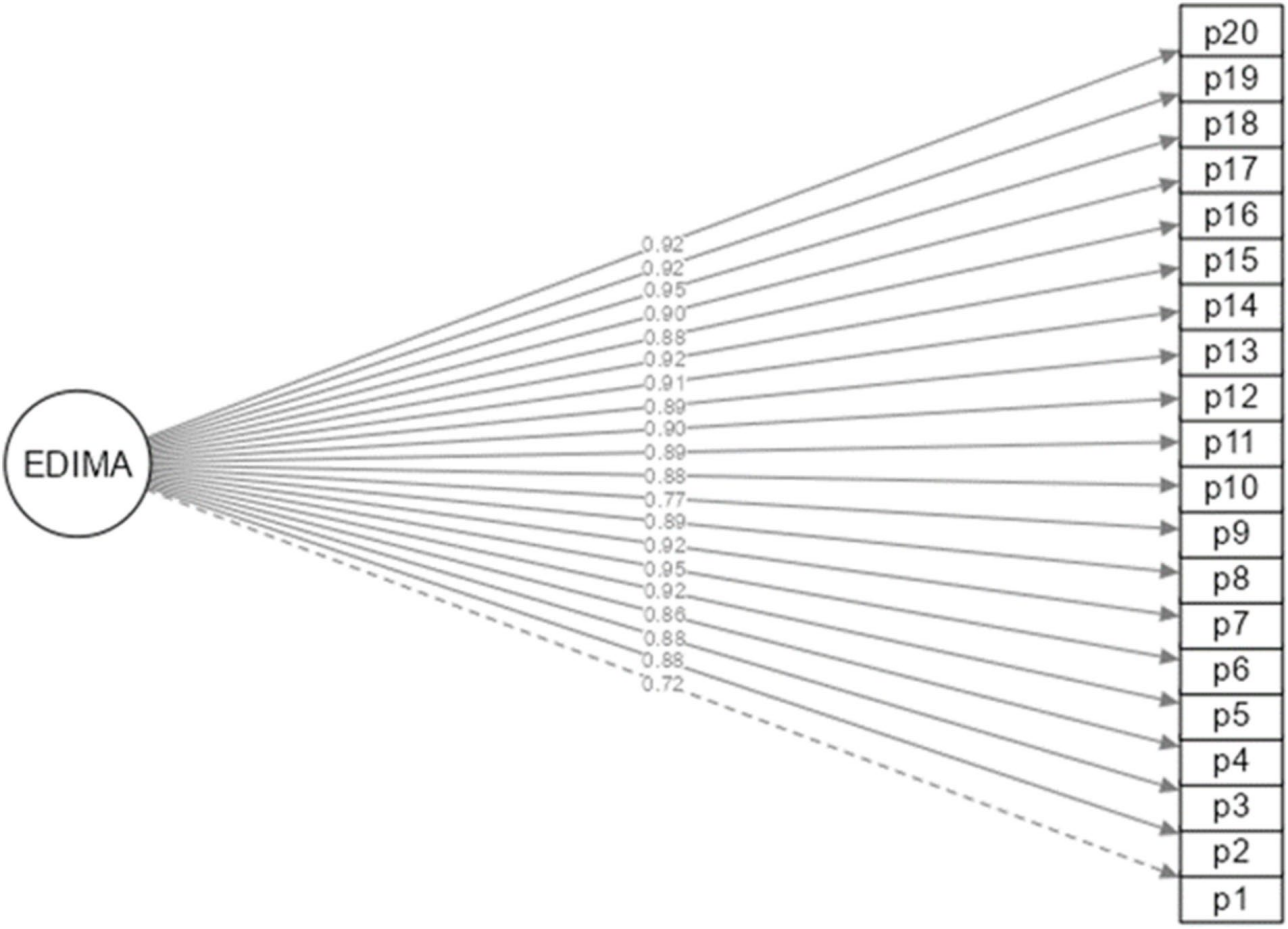
One-factor structure of the EDIMA (*n* = 900).

**Figure 2:**
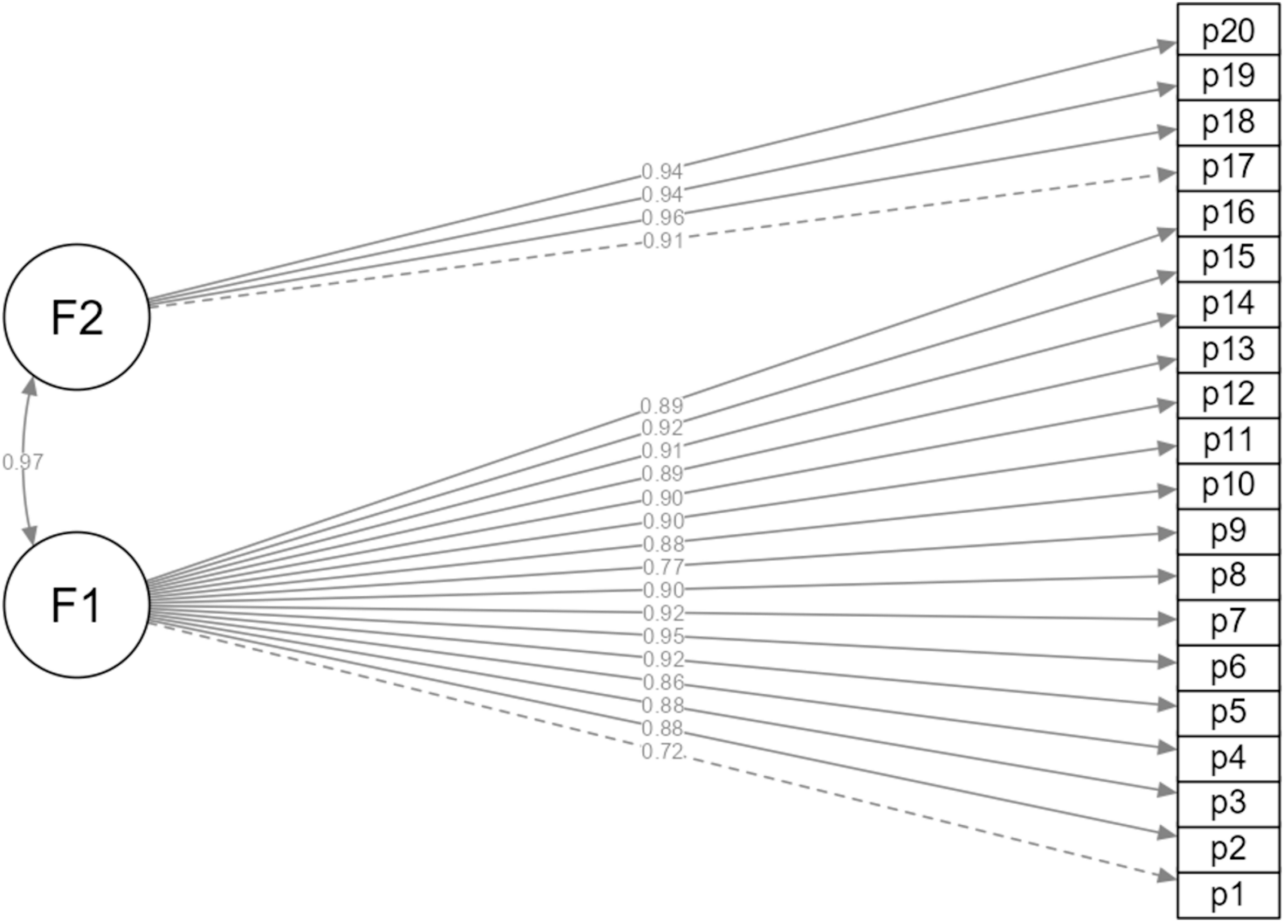
Two-factor structure of the EDIMA (*n* = 900).

Table 3 presents two models of the EDIMA instrument, the first revealing the structure composed of one factor with optimal indicators (CFI = 0.995; TLI = 0.994; SRMR = 0.075; RMSEA = 0.059). Likewise, the original two-factor structure obtained adequate goodness-of-fit indices (CFI = 0.995; TLI = 0.994; SRMR = 0.074; RMSEA = 0.058). In both models, a minimum of *λ* = 0.72 and a maximum of *λ* = 0.95 and *λ* = 0.96 were loaded for the one-dimensional model and for the two-factor structure, respectively (Figures 1, 2).

**Table 3:** Goodness-of-fit indices in the one-factor and two-factor models proposed in the EDIMA.

Model	Goodness of fit index	General
		(*N* = 900)
01 Factor	*χ* ^2^	694
	CFI	0.995
	TLI	0.994
	SRMR	0.075
	RMSEA	0.059
02 Factors	*χ* ^2^	684
	CFI	0.995
	TLI	0.994
	SRMR	0.074
	RMSEA	0.058

X2(df) for model versus baseline. CFI, comparative fit index; TLI, Tucker-Lewis index; SRMR, standardized root mean squared residual; RMSEA, root mean squared error of approximation.

### 3.4 Reliability

The EDIMA instrument as a unidimensional structure presented excellent overall reliability with a *ω* = 0.947 (0.942–0.952). Likewise, as a two-factor model there was good reliability with McDonald’s ω above 0.8 for both factors, with ranges from 0.85 to 0.94 (see Table 4).

**Table 4:** Reliability estimation.

	*ω* de McDonald
Global	0.947 (0.942–0.952)
F1	0.936 (0.93–0.94)
F2	0.872 (0.858–0.885)

95% CI; upper and lower bound.

### 3.5 Measurement invariance

The results of the MIMIC models confirmed invariance by age and sex (Table 5). The ΔCFI and ΔRMSEA values were ≤ 0.01 for both groups. Furthermore, across age, the CFI and TLI values reported a value = 0.99, while SRMR and RMSEA were always <0.10. Likewise, for the sex group, the CFI and TLI values remained robust (0.99) although the RMSEA was high. This discrepancy suggests minor deviations in model fit for this subgroup, but does not undermine overall factorial validity given strong support from other fit indices.”

**Table 5:** Goodness of fit of the MIMIC models for the EDIMA.

Covariates	Model	CFI	TLI	RMSEA	SRMR	Δ CFI	Δ TLI	Δ MRSEA
Age	Invariant intercept MIMIC	0.996	0.994	0.031	0.017	*	*	*
	Satured MIMIC	0.995	0.994	0.028	0.019	−0.001	0.000	−0.003
Sex	Invariant intercept MIMIC	0.999	0.999	0.119	0.130	*	*	*
	Satured MIMIC	0.999	0.999	0.125	0.130	0.000	0.000	0.006

CFI, comparative fit index; TLI, Tucker-Lewis index; SRMR, standardized root mean squared residual; RMSEA, root mean squared error of approximation; ΔCFI, change in comparative fit index between models; ΔTLI, change in Tucker-Lewis index between models; ΔRMSEA, change in root mean square error of approximation between models.

## 4 Discussion

The purpose of the study was to analyze the psychometric properties of the EDIMA with a population of adolescents from different representative regions of Peru (coast, highlands and jungle). The Peruvian version of the EDIMA reported adequate indicators of structural validity and acceptable reliability. Likewise, the EDIMA presents an equivalent performance when making comparisons between age groups and sex.

The Peruvian version of the EDIMA reports psychometric properties similar to those revealed in the original EDIMA conducted with Spanish adolescents (Penado et al., 2019). In both studies, the factorial structure of the EDIMA confirmed its two factors, to assess the Dissemination of Intimate Images among Adolescents through the 20 items proposed in the instrument. For example, in the Spanish validation, fit indices similar to the Peruvian version were reported with values above 0.95 (Penado et al., 2019). Furthermore, with respect to the factor weights of the Peruvian version of the EDIMA they were higher with 0.72 in the first factor and slightly lower with 0.91 in the second factor given that in the original Spanish proposal values of 0.65 and 0.96 were reported, respectively, (Penado et al., 2019). Likewise, in our results and in the original study developed by Penado et al. (2019), a correlation higher than 0.9 was found between both factors. However, we consider that both factors are necessary due to their implications and motivations, for example, the first factor collects information on sexting behavior via cell phone or messaging services that could be motivated by desires for intimacy; while the second factor includes the publication in social networks of this type of content, which would imply massive exposure causing harm and victimization. In addition, we show that the two-factor model presents a better fit to the data according to the RMSEA and SRMR fit indices compared to the unidimensional model.

The reliability of the EDIMA in the sample of Peruvian adolescents was acceptable. This result is consistent with the original study which reported an *α* = 0.976 in its two-factor model (Penado et al., 2019). Likewise, in Nigerian adolescent students the EDIMA has reported a lower internal consistency with a Cronbach’s alpha equal 0.81 (Adigun, 2020a). In that sense, the EDIMA can be applied in adolescents from different regions of Peru by professionals in psychology in health centers or academic schools to obtain consistent, accurate and reliable results in estimates of the dissemination of intimate images among adolescents.

Our results revealed that the EDIMA scale exhibits evidence of measurement invariance across age and sex groups, suggesting that the scale consistently measures the same underlying construct in adolescents, regardless of their age and sex. Although the RMSEA for sex invariance was high, this result should be interpreted with caution. RMSEA is known to be sensitive to sample size and model complexity, which could explain this deviation. Furthermore, results from other indices indicated excellent model fit, supporting the overall validity of the model across all groups in our study (Chen, 2007; Byrne, 2010). Likewise, our results could not be compared with other studies that have assessed the measurement invariance of the EDIMA, the findings represent the first evidence of its comparable functioning across these groups. Likewise, other studies frequently use EDIMA to assess and compare groups, without sufficient evidence to perform these analyses (Molla-Esparza et al., 2023). These studies use EDIMA and assume its equal performance regardless of the demographic diversity of the sample and do not repair the invariance that could occur in the different groups (Adigun, 2020b; Koletić, 2022). In that sense, the evidence of invariance across groups indicates that the scale is able to reliably capture the dissemination of intimate images in adolescent males and females, ensuring that the results obtained are comparable and equitable across different ages from 12 to 17 years (Meredith, 1993). This consistency is fundamental for the application of the scale in longitudinal and comparative studies without risk of gender bias (Widaman et al., 2010).

### 4.1 Implications

Our findings favor and expand the psychometric evidence on the utility of the scale, allowing researchers and practitioners to be confident in the consistency of the results obtained from the Peruvian version of the EDIMA. In this sense, it should be noted that the psychometric properties evaluated in this study are specific characteristics of the Peruvian population. Therefore, although the scale may have applications in various disciplines such as psychology, sociology, law and public health, the results obtained are more relevant to the Peruvian adolescent population. This facilitates the approach to the phenomenon from a multidisciplinary perspective, allowing for a comprehensive analysis that considers both the individual dimensions of behavior and the social, legal and health contexts. Future studies could address prevalence or associated factors using the validated scale to explore these aspects.

The average scores obtained in the EDIMA suggest that both men and women from different regions of Peru are exposed to similar risks in relation to the dissemination of intimate images. Therefore, future studies with the purpose of developing intervention and prevention strategies should be designed in an equitable manner, emphasizing the needs and risks of adolescents in a comprehensive sexual education that includes responsible use and privacy through electronic devices as well as the consequences of dissemination on social networks.

Likewise, a reliable instrument such as EDIMA is essential for government institutions in the task of addressing this complex phenomenon. For example, estimates of this problem could lay the groundwork for the implementation of specific actions such as policies and laws that specifically address the non-consensual dissemination of intimate images between adolescents, with clear limits and legal consequences. In this way, the safety and protection of adolescents is guaranteed, minimizing the emotional damage associated with this type of dissemination.

### 4.2 Strengths and limitations

This study represents the first analysis providing psychometric evidence of the Diffusion of Intimate Images Among Adolescents Scale (EDIMA) as a reliable instrument to assess the diffusion of intimate images among Peruvian adolescents. It is important to note that the study specifically focuses on Peruvian adolescents, which is essential to understanding the diffusion of intimate images in this particular demographic group. Additionally, the confirmation of the two-factor structure through structural analysis demonstrates a rigorous approach to scale validation, supporting the robustness of the measurements. Although a two-factor structure of the EDIMA was confirmed and reflects the original proposal of Penado et al. (2019), a high correlation (*r* = 0.97) was observed between the factors. This suggests that the factors share a large proportion of variance, raising the possibility of considering a unidimensional structure in future research. In addition, this is the first study to show evidence of invariance by age group and sex, highlighting the instrument’s ability to perform equivalently across age and sex group comparisons, thus demonstrating its utility at various stages of adolescent development. However, certain limitations should be considered when interpreting the findings. First, the selection of participants through non-probability sampling could introduce biases and limit the generalization of the results to the entire adolescent population of Peru. Nevertheless, our study included 900 students from the coastal, highland, and jungle regions of Peru, providing a broad national geographical representation. On the other hand, the results showing high factor loadings may indicate a possible partial overlap of the items. However, the multicollinearity analysis and the model fit indices did not indicate severe issues. Consequently, for future research, we recommend a thorough review of the items to eliminate those with potential conceptual redundancy. In addition, we also consider it pertinent that future studies apply a multigroup analysis method to assess invariance at different levels (configural, metric, scalar, and strict), and generate a deeper detail of measurement equivalence between groups. This approach would provide a more accurate picture of age and sex invariance, strengthening the implications of the study for its application at different stages of adolescent development. Likewise, a criterion validity analysis was not performed due to the absence of instruments with solid psychometric properties for this particular population of adolescents living in different regions of Peru, with their own cultural and linguistic characteristics. This limitation prevents us from assessing how EIDMA relates to other related measures, which could strengthen the understanding of intimate image dissemination behavior. Future research should explore the possibility of conducting a criterion validity analysis using scales that assess related constructs such as sexting to expand the evidence of scale validity.

## 5 Conclusion

In conclusion, the EDIMA is a valid and reliable instrument for estimating the dissemination of intimate images in Peruvian adolescents living in the coast, mountains and jungle of Peru. The Peruvian version of the EDIMA with 20 items has shown validity in its two-factor model and acceptable reliability. It also shows evidence of invariance across age and sex, and in this sense, it can be applied in these populations.

## Data Availability

The raw data supporting the conclusions of this article will be made available by the authors, without undue reservation.
