# Supplementary material for: Validation and adaptation of the diffusion of intimate images among adolescents (EDIMA) scale in adolescents from Peru
Source: Front Psychol. 2024 Dec 16;15:1399423. doi: 10.3389/fpsyg.2024.1399423 (PMC11695780; doi:10.3389/fpsyg.2024.1399423)
Supplement: Supplementary file 1 [file Table_1.DOCX]

Supplementary Material

| **EDIMA** (Escala de Difusión de Imágenes Íntimas entre Adolescentes versión en español)  A continuación, te presentamos una serie de conductas que se realizan a través de las **redes sociales** y que tienen que ver con la **difusión de imágenes de contenido íntimo y/o personal**. Por favor señala en cada uno de los ítems que te presentamos **la frecuencia con la que has realizado cada uno de los comportamientos** de acuerdo a la siguiente escala: | | | | | | | |
| --- | --- | --- | --- | --- | --- | --- | --- |
| **1. Nunca** | **2. Rara vez**  (1 o 2 veces al mes) | **3. Ocasionalmente**  (2 o 3 veces al mes) | **4.   A menudo**  (2 o 3 veces por semana) | **5. Frecuentemente**  Todos los días | | | |
| Los datos recogidos son totalmente **CONFIDENCIALES** y ni los profesores ni tus padres tendrán acceso a ellos por lo que te pedimos que seas **TOTALMENTE SINCERO** en tus manifestaciones | | | | | | | |
| A través del teléfono celular (chats) | | | | | | | |
| 1.    He **recibido** *imágenes/videos insinuantes o provocativas* | | | 1 | 2 | 3 | 4 | 5 |
| 2.    He **enviado** *imágenes/videos insinuantes o provocativas* *míos* | | | 1 | 2 | 3 | 4 | 5 |
| 3.    He **reenviado** *imágenes/videos insinuantes o provocativas* | | | 1 | 2 | 3 | 4 | 5 |
| 4.    He **pedido** *imágenes/videos insinuantes o provocativas* | | | 1 | 2 | 3 | 4 | 5 |
| 5.    He **recibido** *imágenes/videos íntimos o provocativos* de mi **pareja** | | | 1 | 2 | 3 | 4 | 5 |
| 6.    He **enviado** *imágenes/videos íntimos o provocativos míos* a mi **pareja** | | | 1 | 2 | 3 | 4 | 5 |
| 7.    He **reenviado** *imágenes/videos íntimos* ***de mi pareja*** *sin su permiso* a una **tercera persona** | | | 1 | 2 | 3 | 4 | 5 |
| 8.    He **pedido** *imágenes/videos insinuantes o provocativas* a mi pareja | | | 1 | 2 | 3 | 4 | 5 |
| 9.    He **recibido** imágenes/vídeos íntimos o provocativos de un **desconocido** | | | 1 | 2 | 3 | 4 | 5 |
| 10. He **enviado** imágenes/videos íntimos o provocativos míos a un **desconocido** | | | 1 | 2 | 3 | 4 | 5 |
| 11. He **reenviado** imágenes/videos íntimos de un desconocido *sin su permiso* a una tercera persona | | | 1 | 2 | 3 | 4 | 5 |
| 12. He **pedido** *imágenes/videos insinuantes o provocativas* a un desconocido | | | 1 | 2 | 3 | 4 | 5 |
| 13. He **recibido** imágenes/videos íntimos o provocativos de un **amigo/a** o conocido/a | | | 1 | 2 | 3 | 4 | 5 |
| 14. He **enviado** imágenes/videos íntimos o provocativos míos a un **amigo/a** o conocido/a | | | 1 | 2 | 3 | 4 | 5 |
| 15. He **reenviado** imágenes/videos íntimos de un amigo/a o conocido/a una tercera persona sin su permiso | | | 1 | 2 | 3 | 4 | 5 |
| 16. He **pedido** *imágenes/videos insinuantes o provocativas* a un **amigo/a** o conocido/ | | | 1 | 2 | 3 | 4 | 5 |
| En las **redes sociales** (Instagram, Facebook) | | | | | | | |
| 17. He publicado imágenes/vídeos míos atrevidas o provocativas | | | 1 | 2 | 3 | 4 | 5 |
| 18. He publicado imágenes/vídeos atrevidas o provocativas de mi pareja sin su permiso | | | 1 | 2 | 3 | 4 | 5 |
| 19. He publicado imágenes/vídeos atrevidas o provocativas de un desconocido sin su permiso | | | 1 | 2 | 3 | 4 | 5 |
| 20. He publicado imágenes/vídeos atrevidas o provocativas de un conocido/a o amigo/a sin su permiso | | | 1 | 2 | 3 | 4 | 5 |
